# Assessment of Quality of Life Among Patients With Primary Hypothyroidism: A Case-Control Study

**DOI:** 10.7759/cureus.29947

**Published:** 2022-10-05

**Authors:** Ranya Ghamri, Raghad Babaker, Salwan Ezzat, Haya Alsaedi, Muruj Alkhamisi, Ragad Arbaein, Rahaf Alyahya, Shahad Fayraq, Sultan Alamri

**Affiliations:** 1 Family Medicine, King Abdulaziz University, Jeddah, SAU; 2 Medical School, King Abdulaziz University, Jeddah, SAU

**Keywords:** saudi arabia, physical health, mental health, hypothyroidism, quality of life

## Abstract

Introduction

Hypothyroidism is a globally prevalent condition with a huge impact on health. It has been demonstrated that hypothyroidism is associated with negative health outcomes that have a significant impact on quality of life. The aim of this study is to assess the relative significance of various parts of quality of life satisfaction in adult patients with hypothyroidism. This study has never been done in Saudi Arabia. No previous research was conducted in our region to highlight the quality of life of these patients. From this, it is very important to acknowledge the impact of hypothyroidism on quality of life, to help improve their lives and reduce the burden of the disease.

Method

This is a case-control study conducted on patients with hypothyroidism presented to the family medicine clinic at King Abdulaziz University Hospital. The participants were divided into two groups: patients with primary hypothyroidism (cases) and euthyroid subjects of the same age without chronic illnesses (control group). We used the Short Form-12 questionnaire (SF-12) and a sociodemographic questionnaire as a tool to assess the quality of life.

Results

Compared to healthy euthyroid controls, patients with hypothyroidism had a significantly reduced quality of life regarding their physical (p<0.001) aspect, with mental health not being affected. Smoking, female gender, and BMI showed significant associations with their mental health status (p=0.021, p=0.001, and p=0.045) respectively.

Conclusions

Regarding the results of the current study, there is a reduced level of physical health in patients with hypothyroidism than in healthy controls, which adversely affects their QoL. Smoking, gender, and BMI were associated with lower mental health among patients with hypothyroidism, which adversely affected their quality of life. Health practices should include assessment of the quality of life, monitoring by specialized physicians, and educational programs for these patients.

## Introduction

Hypothyroidism is a common condition worldwide with a severe impact on health [1]. It is a lifelong chronic condition whereby the thyroid hormone is produced in insufficient amounts causing multiple symptoms, such as slow movements, slow metabolism, cold intolerance, fatigue, hoarseness, weight gain, and constipation. There could also be defects in cognitive function, such as decreased attention and poor memory [2]. Hypothyroidism is also linked to a wide range of psychiatric disorders, especially depression and paranoid disorders [2].

Hypothyroidism is more prevalent in women than in men, and the disease is strongly associated with increasing age [3]. The prevalence of hypothyroidism is 4-5%, hence making it a global endocrine disorder [4]. A study conducted among 567 participants in Saudi Arabia showed a 15.9% prevalence of subclinical hypothyroidism [5].

It has been acknowledged that hypothyroidism is associated with negative health outcomes (faint to extreme) [6]. It has a considerable effect on the quality of life (QoL) (McMillan et al., 2008) [2]. The World Health Organization (WHO) defines QoL as an individual's perception of their position in life in the context of the culture and value systems in which they live and in relation to their goals, expectations, standards, and concerns [7]. Health-related quality of life (HRQoL) is a useful tool designed to estimate an individual’s sense of the effect of disability on that person’s physical, social, and psychological functioning [8]. 

Previous studies have revealed that HRQoL is frequently reduced in patients with hypothyroidism when compared to the general population [9]. In addition, a reduced QoL remains a concern for several patients with hypothyroidism on levothyroxine (L-T4) medication, as reported in another study [10,11]. A greater effect of hypothyroidism on the QoL of patients with lower mental health levels has been determined [12] and one of the most prevalent psychiatric parameters affected by hypothyroidism is depression in the elderly [13]. An earlier study in a large group of treated patients with hypothyroidism found an inverse relationship between their QoL and body mass index (BMI) [10]. Although such findings are important and should be given more attention, unfortunately, no studies in Saudi Arabia have evaluated QoL in patients with hypothyroidism.

This study aimed to estimate the impact of hypothyroidism on QoL and to explore factors associated with reduced QoL. Such a study has never been conducted in Saudi Arabia. Although several studies have focused on the prevalence and risk factors of hypothyroidism, no research has been conducted to highlight the QoL of these patients. It is important to acknowledge the impact of hypothyroidism on the QoL to help improve the patients’ lives and reduce the burden of the disease.

## Materials and methods

This case-control study included patients with hypothyroidism who visited the Family Medicine Clinic at King Abdulaziz University Hospital in Jeddah, Saudi Arabia from January 2020 to March 2022. The sample size was calculated using an online Epi calculator. Based on the assumption that the proportion exposed in the control group is 50%, and a power of 80% at the 95% confidence interval, the calculated sample size per group was 100. A total of 209 patients who met the inclusion criteria were included in this study. The sample was divided into two groups: patients with primary hypothyroidism (cases) and euthyroid subjects of the same age without chronic illnesses (control group). Neither of the study groups was subjected to any intervention or invasive procedures.

For all participants, the inclusion criteria were age between 18-65 years and confirmed hypothyroidism. The exclusion criterion was known pregnancy in the last 12 weeks. We also excluded patients who were unable to complete the questionnaire because of cognitive disorders.

The Short-Form 12-point Health Survey (SF-12) is a valid alternative to SF-36 for use in large surveys of general and specific populations [14,15]. The Cronbach's alpha value (0.8) reflected satisfactory internal consistency reliability of SF-12. All SF-12 items were obtained from SF-36. It includes eight dimensions: general mental health, physical functioning, bodily pain, general health perception, vitality, social functioning, role limitations due to physical health problems, and role limitations due to emotional health problems. The scores per scale range from 0 to 100, with the highest score indicating the best state of health. The eight dimensions were grouped into physical and mental components. The physical component included the following questions: general health status, moderate activities, limitation in the ability to climb several flights of stairs, reduced accomplishments due to physical health, pain interfering with activity, and limitation in the kind of work due to physical health. Mental health was defined as having accomplished less due to emotional problems, working less carefully, feeling calm and peaceful, feeling downhearted and “blue,” and lacking in energy.

Participants were asked to complete a questionnaire (Short Form-12 (SF-12) questionnaire). The data sheet included age, sex, nationality, marital status, social status, smoking, physical activity level, other chronic diseases, BMI, interference of physical and emotional health with the individual’s social activities, and accomplishment of daily activities.

Ethical issues

Ethical approval was obtained from the Biomedical Research Ethics Committee, Faculty of Medicine, King Abdulaziz University, Jeddah, Kingdom of Saudi Arabia on September 15, 2020 (Reference No 474-02).

Statistical analysis

Statistical analysis was conducted using SPSS statistics v. 20.0 software (IBM Corp., Armonk, NY). The Shapiro-Wilk test was used to test the normality of the study samples. Simple frequency tables, cross-tabulations, and percentages were also used and for the descriptive statistics, the median (25th, 50th, 75th percentiles) was used. The chi-squared test was used to test and describe the relationship between two categorized variables. The non-parametric Mann-Whitney test analysis was used to determine significant differences between the two groups. Binomial logistic regression was used to test the predictors of binary outcome variables. A p-level of <0.05 was considered statistically significant.

## Results

In total, 209 men and women met the eligibility criteria. The numbers of participants in the case and control groups were 99 and 110, respectively. All participants completed the SF-12 questionnaire. Our results are presented using median instead of mean because the data were not normally distributed. Table 1 shows the sociodemographic profile of the participants. The median height (cm) was 163 cm, weight (kg) was 74 kg, and BMI was 27.34. The majority of our sample (66.5%) was women. Most of the participants (34.4%) were aged 18-28 years, while fewer (17.2%) were aged between 51-60 years. Most of the participants (89%) were natives of Saudi Arabia. More than half of the participants (62.2%) were married. Obese, overweight, normal weight and underweight individuals accounted for 34.4%, 30.1%, 32.5%, and 2.9% of the sample, respectively.

**Table 1:** Comparison of sociodemographic profile of the participants (cases and controls), n=209 BMI, body mass index

Variable		Control	Case	P value
Gender	Male	55.7%	44.3%	0.526
	Female	51.1%	48.9%	
Age	18–28	77.5%	22.5%	<0.001
	29–39	47.4%	52.6%	
	40–50	40.6%	59.4%	
	51–60	30.6%	69.4%	
Nationality	Saudi	54.3%	45.7%	0.169
	Non-Saudi	39.1%	60.9%	
Marital status	Single	74.2%	25.8%	<0.001
	Married	46.2%	53.8%	
	Widowed/ divorced/ separated	23.5%	76.5%	
BMI	Under weight	83.3%	16.7%	<0.001
	Normal	76.5%	23.5%	
	Overweight	44.4%	55.6%	
	Obese	34.7%	65.3%	
Income	I do not have enough money to pay my expenses.	54.5%	45.5%	0.064
	I only have enough money to pay my expenses but no cash left.	42.1%	57.9%	
	I usually have some extra money left over.	59.5%	40.5%	

**Table 2:** Comparison of the mental and physical health of the cases and controls using the SF-12 The mental and physical health of the whole sample is shown in Table 2. A score below the 50th percentile (the median) indicates poor QoL. Scores above the 50th percentile indicated excellent QoL. It was found that patients with hypothyroidism had poor physical health when compared to healthy euthyroid control, while mental health was less affected than physical health.

			Control	Case	P value
Mental health	≤ 50 percentile	Count	52	54	0.294
		Percent	49.1%	50.9%	
	> 50 percentile	Count	58	45	
		Percent	56.3%	43.7%	
Physical health	≤ 50 percentile	Count	47	70	<0.001
		Percent	40.2%	59.8%	
	> 50 percentile	Count	63	29	
		Percent	68.5%	31.5%	

**Table 3:** Factors associated with the mental health of the study participants: regression analysis This table illustrates the relationship between the mental health of the cases and other variables affecting mental health. We found that smoking, gender, and BMI significantly affected mental health. No significant relationships were found between any of the variables and physical health. BMI, body mass index; KAUH, King Abdulaziz University Hospital; OR: odds ratio; CI: confidence interval

Variable	P-value	OR	95% CI	
			Lower	Upper
Gender				
Male		Ref		
Female	0.001	0.020	0.002	0.187
Age				
18–28		Ref		
29–39	0.295	4.346	0.279	67.806
40–50	0.381	3.556	0.208	60.735
Nationality				
Saudi		Ref		
Non-Saudi	0.748	1.419	0.168	12.023
Marital status				
Single		Ref		
Married	0.642	0.585	0.061	5.626
Widowed/Divorced/Separated	0.136	0.069	0.002	2.330
BMI	0.045	0.870	0.758	0.997
Financial situation at the end of the month				
Not enough to pay my expenses		Ref		
Enough to pay my expenses but no cash left	0.344	3.910	0.232	65.979
Some extra money left over	0.156	8.286	0.447	153.651
In the case of hypothyroidism, when was the diagnosis made?				
Less than 5 years		Ref		
5–10 years	0.501	1.849	0.308	11.089
11–15 years	0.989	0.0982	0.078	12.319
>15 years	0.170	5.329	0.487	58.259
Diagnosis made at KAUH		Ref		
Diagnosis made at another hospital	0.036	4.274	1.097	16.655
Use levothyroxine as a treatment	0.999	-	-	-
Committed to the medication	0.692	0.519	0.020	13.369
Committed to regular follow up	0.358	2.549	0.347	18.719
Smoking	0.021	0.100	0.014	0.704
Family history of hypothyroidism	460	0.581	0.137	2.456

## Discussion

Our study was designed to assess the QoL of patients with hypothyroidism and compare it with that of normal individuals. The results showed that physical health was significantly affected related to the QoL in patients with hypothyroidism when compared to the control group, while mental health was less impacted than physical health. The regression analysis showed that only smoking and gender were significant factors influencing mental health among these patients. 

A compatible study revealed that HRQoL was affected by hypothyroidism [16]. Another study that evaluated the potential association between hypothyroidism and QoL in relation to BMI, revealed that patients with hypothyroidism have impaired QoL and experienced more problems with physical functioning [10]. In India, in 2018, Shivaprasad studied the QoL of patients with hypothyroidism and found that these individuals had lower total QoL scores, especially concerning their physical health component [9]. These findings correspond to our own results. Similarly, a study conducted in Italy in 2021 found no correlation between QoL measures and anxiety symptoms [17].

According to our results, mental health was not majorly affected. Interestingly this finding was unexpected. One possible implication of this is that we are a private community, and as result, one might encounter many difficulties while asking patients specifically about mental health-related questions and found that there is little awareness of mental health issues. In contrast to earlier findings, a study that assessed thyroid dysfunction and HRQoL found higher levels of depression, anxiety, and stress symptoms, as well as a worse HRQoL among those with hypothyroidism [18]. Our outcome is contrary to that of Winther et al. (2016) who studied the QoL of patients with hypothyroidism and found that these individuals had lower total QoL scores, particularly regarding the mental health component [12]. Additionally, the findings of the current study do not support the results found by Bathla et al. (2016), who concluded that depression and anxiety-related symptoms were significantly more prevalent in patients with hypothyroidism than in healthy controls [19]. Furthermore, in 2020, Han et al. observed that patients with hypothyroidism had a better HRQoL than euthyroid individuals in the Korean community [20]. These findings contradict those of this study. The small sample size of patients with hypothyroidism may explain these discrepancies. In addition, only 10 patients with hypothyroidism were included in the study, with the remaining 987 euthyroid controls. Another possible reason is that the methods used to assess QoL in each study differed [18].

Regarding BMI, chronically ill obese patients are known to have lower QoL [21]. However, our study showed a significant relationship between higher BMI and decreased QoL in patients with hypothyroidism. Moreover, a study was conducted to evaluate QoL in relation to BMI in patients with primary hypothyroidism, using the SF-36 questionnaire. This study revealed that QOL decreased with increasing body weight which matched the results of our study as well [10]. 

In general, smoking negatively impacts a person’s mental health, causing them to experience depression, anxiety, and stress [22]. Additionally, there is evidence that smoking cessation is beneficial for overall mental health, as discussed in a previous study [22]. The current study concluded that an individual's overall mental health is strongly influenced by two major factors, one of them being smoking. This finding is contrary to those of a previous study that suggested that tobacco consumption had no connection with the HRQoL in women with hypothyroidism [16]. In concordance with the findings of Morón-Diaz et al. (2021), our data demonstrated an association between QOL and cigarette consumption [23].

Hypothyroidism is one of the most common thyroid disorders, particularly in women [24]. In a study done in Spain, it has been demonstrated that women who undergo treatment for hypothyroidism have markedly poorer mental and physical health than their general population [16]. Due to the strong correlation between the female gender and thyroid disease with depression prevalence, it was not an unexpected outcome that their Qol turned out to be worse than euthyroid women [16,25].

This is the first study to identify the variations in QoL in patients with hypothyroidism and normal individuals in the Saudi population. Most studies have focused only on the prevalence, risk factors, and treatment satisfaction of patients with hypothyroidism. Moreover, the size of our sample is larger than that in prior studies. The scope of this study was limited, in that the number of male and female patients was unequal. An additional uncontrollable factor is the possibility that many of the participants involved in the study had other comorbidities that might impair their QoL aside from hypothyroidism alone. Furthermore, this could also be influenced by an unknown history of medications, which might have led to inadequate treatment effects in the hypothyroid group.

## Conclusions

Based on the results of the present study, there is a reduced level of physical health in patients with hypothyroidism than in healthy controls, which adversely affects their QoL. One of the more significant findings of this study is that smoking and the female gender were associated with worse mental health among patients with hypothyroidism.

These findings suggest that QoL in patients with hypothyroidism needs more attention, and health practices should include monitoring by specialist physicians and educational programs.
